# Expediting treatments in the 21st century: orphan drugs and accelerated approvals

**DOI:** 10.1186/s13023-024-03398-1

**Published:** 2024-11-08

**Authors:** Reuben Domike, G. K. Raju, Jamie Sullivan, Annie Kennedy

**Affiliations:** 1 Light Pharma Inc, Cambridge, Massachusetts USA; 2 M.I.T. Center for Biomedical Innovation, Massachusetts Institute of Technology, Cambridge, Massachusetts USA; 3 University of Prince Edward Island, Charlottetown, PE, Canada; 4 EveryLife Foundation for Rare Diseases, Washington, DC USA

**Keywords:** Orphan drug designation, Accelerated approval, Confirmation trials

## Abstract

**Background:**

In response to activated patient communities’ catalyzation, two significant efforts by the FDA to expedite treatments have now been in place for multiple decades. In 1983, the United States Congress passed the Orphan Drug Act to provide financial incentives for development of drugs for rare diseases. In 1992, partly in response to the HIV epidemic, the FDA implemented Accelerated Approval (AA) to expedite access to promising new therapies to treat serious conditions with unmet medical need based on surrogate marker efficacy while additional clinical data is confirmed.

The uses of these regulatory approaches over time are assessed in this study.

**Methods:**

The following U.S. FDA CDER published lists were used in this analysis: 1. all orphan designations and approvals; 2. all AA and their details updated through December 31, 2022; new molecular entities (NMEs).

**Results:**

Orphan drug designations and approvals have increased several-fold over the past four decades. The largest increase recently has been in therapies targeting oncological diseases (comprised of both oncology and malignant hematology).

Although orphan drug approvals based on NMEs are the minority of orphan drug designations, the count of approved orphan drug NMEs has increased in recent years.

The characteristics of orphan drug approvals show notable differences by disease area with rare diseases and medical genetics (49%) having a relatively large fraction of orphan drug approvals with NMEs compared to the oncological diseases (32%).

Similar to the use of orphan drug designation, oncological disease therapies have been the largest utilizers of AA. Many therapies targeting these diseases address unmet medical need and can leverage surrogate markers that have previously been used in similar trials.

The timings of conversion of AA (confirmed or withdrawn) were assessed and found to be consistent across decades and to have some dependency upon the broad disease area (when assessed by three large groups: HIV conversions were fastest; followed by oncology; followed by all others). By the end of 2022, 98% of the first 105 (approved in 2010 or earlier) AA had been converted to confirmed or withdrawn.

**Conclusions:**

Although the typical timings for AA to be confirmed or withdrawn has not changed significantly over the decades, the disease areas utilizing orphan drug designation and AA have changed significantly over time. Both programs have had increases in their use for therapies targeting oncological diseases.

The re-use of surrogate markers for oncological diseases has been an advantage in a way that may not be scientifically feasible in many other disease areas that have greater differentiation across disease etiology. For non-oncological diseases, applicability of AA is, in part, dependent upon greater focus on characterization and acceptance of novel surrogate markers.

## Background

In 1983, the United States Congress passed the Orphan Drug Act (ODA) to incentivize the development of drugs for rare diseases, defined in the ODA as affecting fewer than 200,000 people in the US. These incentives were primarily financial, to stimulate the biopharmaceutical industry’s interest in developing drugs for the relatively small populations of patients affected by these diseases, many of which are debilitating or life-threatening. Analysis of the last four decades of orphan drug designation indicates that the number of designations granted more than quadrupled between the 1990s and 2010s [10]. There have been many notable successes associated with the ODA. As highlighted by Dr. Goodman, Chief Scientist of the FDA, in 2010: “Many of the 357 approved orphan drugs have been successfully tested on extremely limited numbers of patients, serving as a testament to FDA’s commitment to these patients. This is possible when the best science is flexibly applied and when therapies are truly effective.” [4]

Aligned with the pursuit of development of drugs for rare disease and a marked increase in rare disease product approvals, the EveryLife Foundation for Rare Diseases underscored the public health urgency and continued unmet need when assessing a nearly $1 trillion economic impact of a fraction of the rare disease community [26]. Of the more than 30 million people living with the estimated 10,000 known rare diseases in the United States [5], more than 95% lack an FDA-approved treatment according to the National Center for Advancing Translational Sciences at NIH [13].

In 1992, following an outcry from an activated HIV patient community during the HIV epidemic, the United States Food and Drug Administration (FDA) implemented regulations establishing the Accelerated Approval (AA) option for expedited access to therapies that treat a serious condition and provide a meaningful advantage over available therapies. A meaningful advantage is described FDA is “the drug may demonstrate substantial improvement over existing therapies on one or more clinically significant endpoints, such as substantial treatment effects observed early in clinical development.” [16] The AA program is one of several expedited programs for serious conditions [24]. The AA program enabled the FDA to approve individual therapies based on a surrogate endpoint judged reasonably likely to predict clinical benefit.

Given this level of uncertainty or risk associated with the clinical benefit, as part of the approval process, the sponsor company typically commits to conducting further studies after approval to confirm whether the therapy has clinical benefit. Therapies approved using the AA program using a surrogate endpoint meet FDA’s standards for safety and effectiveness but typically have greater uncertainty in clinical benefit compared to those using traditional regular approval (RA) [6]. This is a trade-off with providing earlier access to therapies for patients with serious conditions where there is an unmet need [3]. In situations of life-threatening diseases, it has previously been noted that many patients, their families, and their doctors have repeatedly communicated their willingness to take on greater uncertainty in exchange for faster access to therapies [25]. Recognizing the importance of the AA program, in 2022 Congress passed numerous legislative provisions. These provisions established an internal cross-center council on the application of AA policies and enabled the FDA to require that further clinical studies be underway by a regulatory approval date and further clarified product withdrawal authorities [9]. The historical trajectories of orphan drug designation, accelerated approvals, and the connection between the two is the focus of this study.

## Methods

The U.S. FDA CDER regularly publishes a list of all new molecular entities (NMEs) approvals entitled “*Compilation of CDER New Molecular Entity (NME) Drug and New Biologic Approvals*”. Similarly, the U.S. FDA publishes a “Orphan Drug Product designation database”. The FDA’s lists updated through December 31, 2022 were used in this analysis.

The U.S. FDA CDER also regularly publishes a list of all AA and their details entitled “*CDER Drug and Biologic Accelerated Approvals Based on a Surrogate Endpoint*”. That list includes status and dates of conversion (to traditional approval or withdrawn). Some AA have not yet been converted. To be consistent with the FDA published list, voluntary withdrawals are not included in the conversion (they continue to be marked in the FDA published list as "Not Yet Converted") perhaps because they are not yet finally settled as permanently withdrawn. The FDA’s list updated through December 31, 2022 was used in this analysis.

For each approval, an indication is stated. By reviewing each approved indication individually, the authors assigned each indication to a disease area.

## Results

**Fig. 1:**
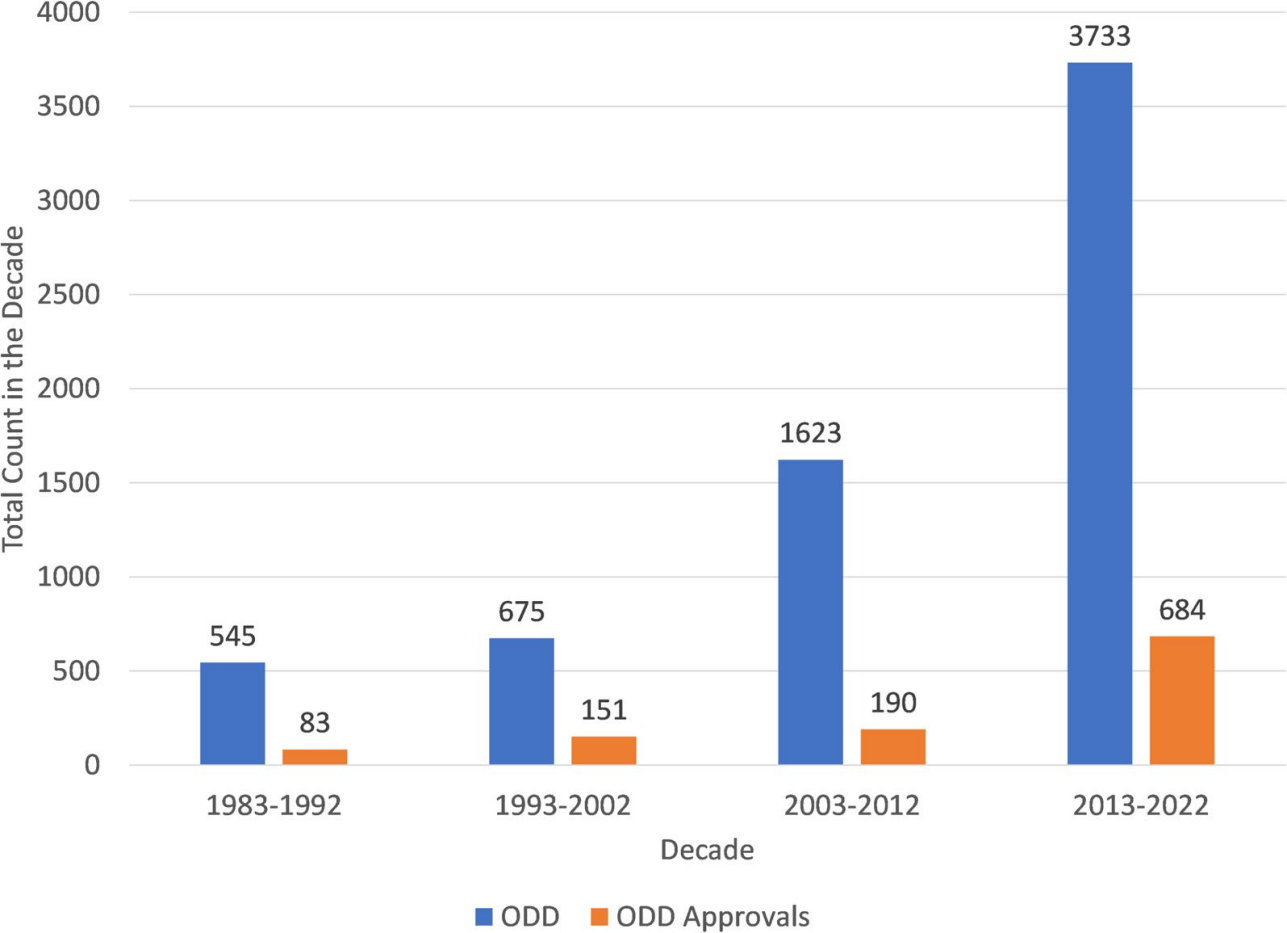
Count of orphan designations and approvals by time period

The timing of all orphan drug designations (ODD) and orphan drug approvals in the U.S. FDA’s relevant list were analyzed starting from the first orphan drug designations in 1983. A summary view of the count of these two categories is presented in Fig. 1. There is some time lag expected between ODD and subsequent approval. However, comprehensively across the decades of data presented in Fig. 1, the vast majority (5468 out of 6576 or 83%) of ODD have not been subsequently approved.

**Fig. 2:**
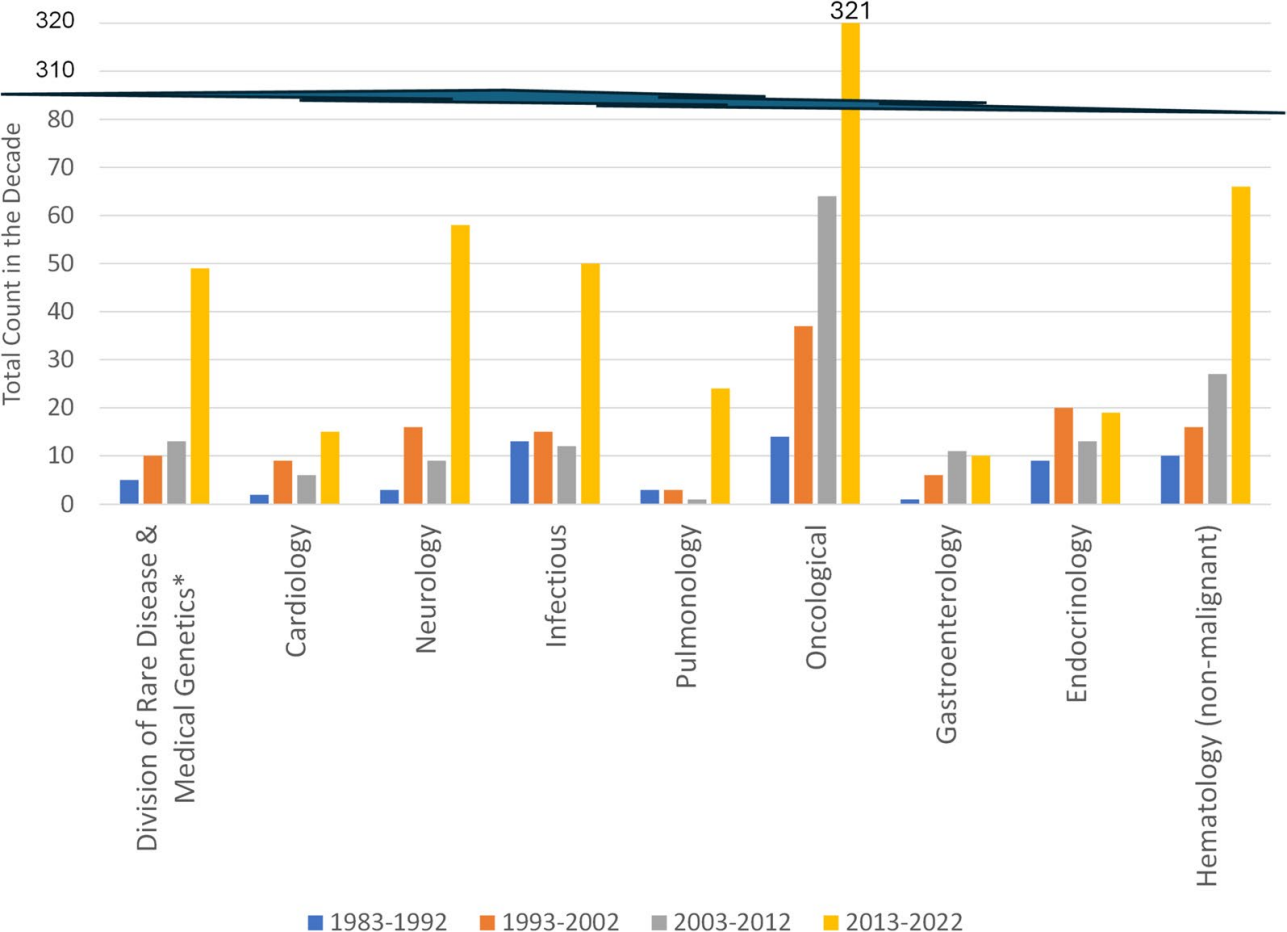
Orphan drug approvals by disease area for those with more than 25 orphan drug approvals

The increase in orphan approvals displayed in Fig. 1 has not been evenly distributed across disease areas. Each approval of an indication using an orphan drug was reviewed and categorized based on the disease intended for treatment. Of the 1108 ODD cumulative approvals displayed in Fig. 1, the disease areas with more than 25 cumulative orphan drug approvals are shown in Fig. 2 in an attempt to observe significant trends over the decades. Amongst those disease areas: 436 (39%) are, in cumulative, oncological diseases, 119 (11%) are non-malignant hematology, 90 (8%) are infectious diseases, and the other 463 (42%) (those of which are not in disease areas with more than 25 total orphan drug approvals are not shown in Fig. 2) are spread over many other disease areas. The disease areas in Fig. 2 are ordered from most frequent in total to least and the grouping of Rare Disease & Medical Genetics (noted with an asterisk) is done to be consistent with the FDA Divisions in 2022. The Rare Diseases & Medical Genetics division within the FDA reviews treatment of rare inborn errors of metabolism and serves as a hub for rare disease drug development across Office of New Drugs [17, 18, 20–23]. The majority of these disease areas have had more ODD approvals in the most recent decades than in the prior three decades combined, indicating significant recent increases in the pursuit of ODD drugs and approvals.

**Fig. 3:**
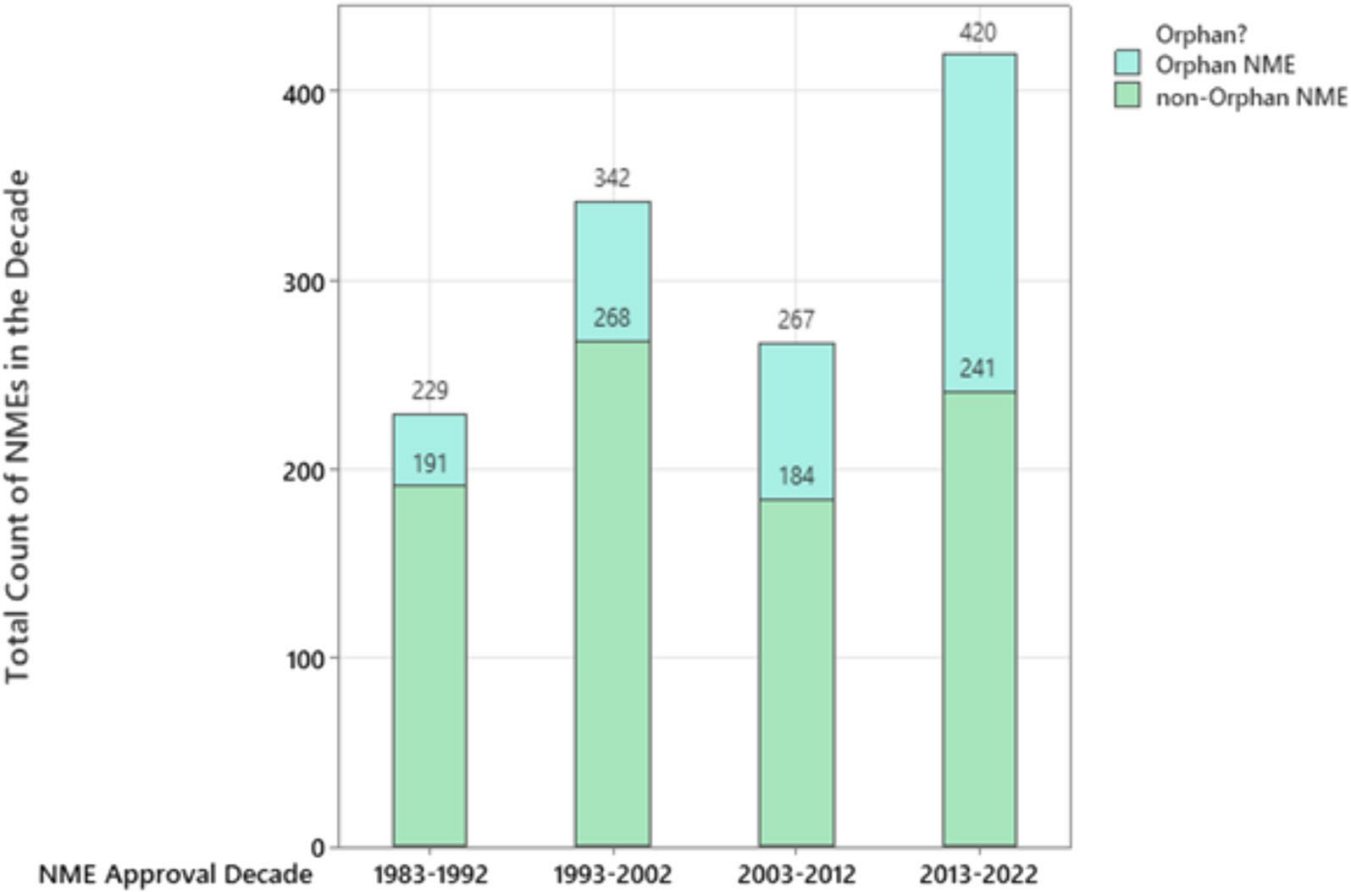
Count of NME approvals segregated based on orphan and non-orphan

One indicator of the recent developments in ODD drugs and approvals is the number of novel therapies available to patients. By integrating the U.S. FDA’s list of orphan drug approvals and the U.S. FDA CDER list of approved NMEs, the quantity of NME approvals in the most recent thirty-eight years was segregated based on orphan designation (or not) and is displayed in Fig. 3. Within Fig. 3, the data labels show cumulative values within the year (e.g. 2013-2022 had 179 orphan NME and 241 non-orphan NME for a total of 420). The number of orphan NME approvals in the most recent decade is significantly higher than in prior decades. Different from orphan NME approvals, the number of non-orphan NME approvals has been relatively stable over the recent two decades.

**Fig. 4:**
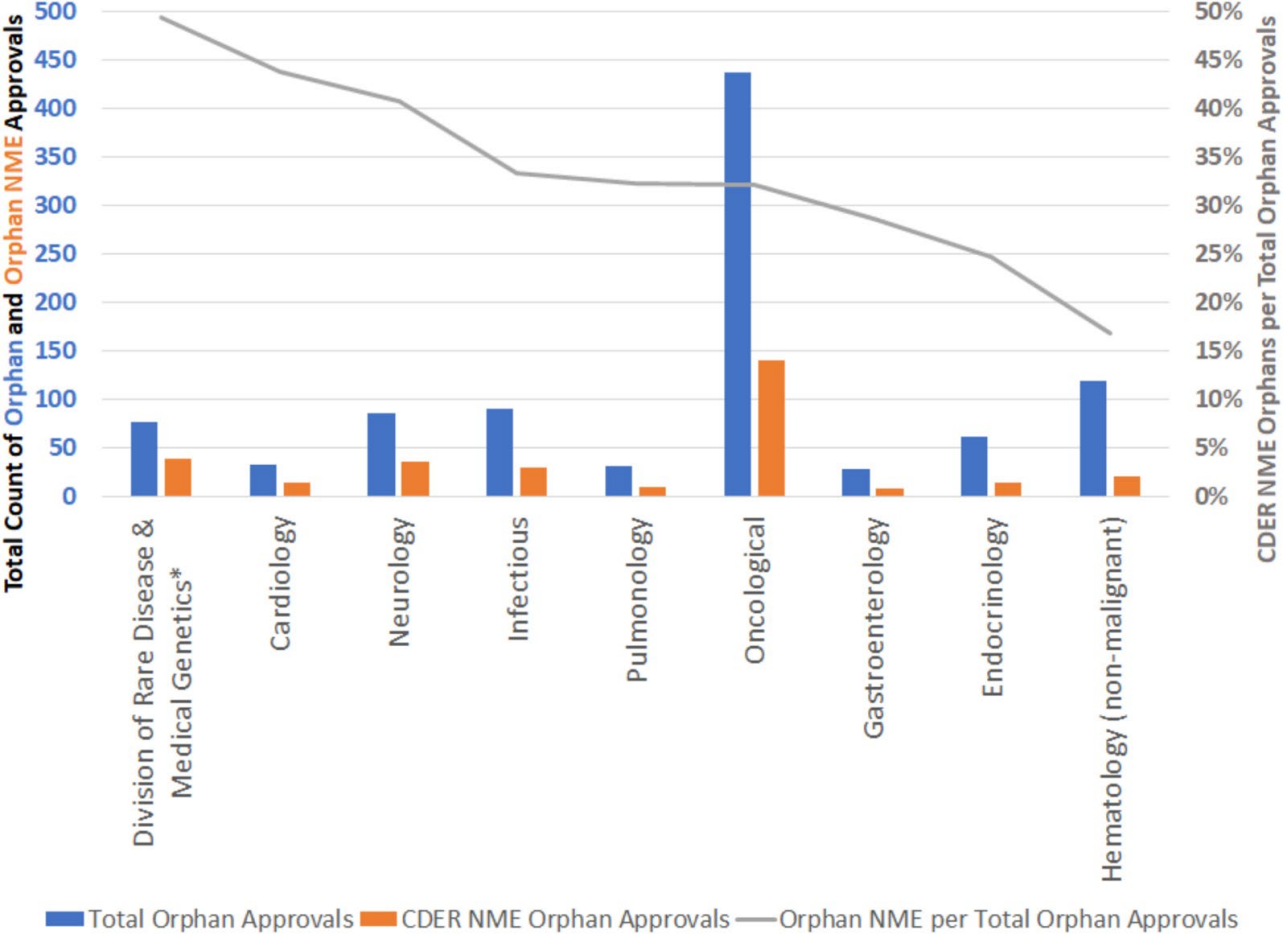
Count of orphan approvals by disease area segregated based on NME designation by CDER

The fraction of orphan drug approvals that were NME is different for different disease areas, as depicted in Fig. 4. The ordering of disease area in Fig. 4 is from most frequent NME to least frequent and the grouping of Rare Disease & Medical Genetics (noted with an asterisk) is done to be consistent with the FDA Divisions in 2022. As a group, diseases that fall within the FDA Division of Rare Disease & Medical Genetics have the highest rate of orphan approvals that are NME. This is aligned with the logic of these rare to ultra-rare diseases (e.g. representing inborn errors of metabolism) being more likely to have relatively differentiated etiologies and therefore not using existing therapies that targeted other diseases.

**Fig. 5:**
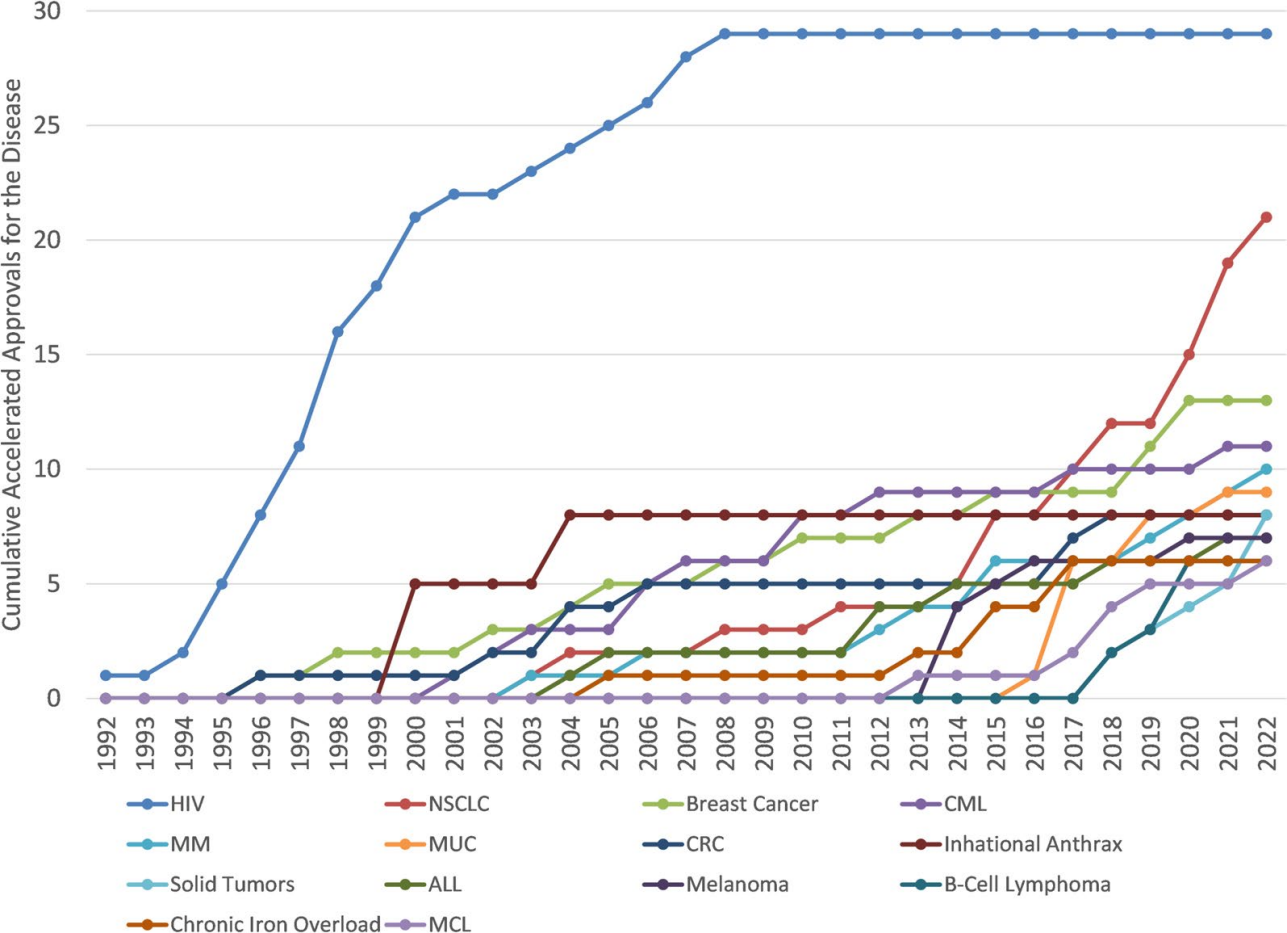
Cumulative AAs for diseases with more than 5 AAs (legend ordered from most to least)

In addition to the orphan designation, accelerated approvals (AAs) were initiated by the FDA in 1992 to enable faster approval and availability based on surrogate markers. Many of the early AAs were for therapies designed to treat HIV. Based on the U.S. FDA CDER’s list of AAs, all diseases with more than five total AAs are shown with their cumulative count of AAs in Fig. 5. Other than HIV and Inhalation Anthrax and Chronic Iron Overload, all other diseases with more than five AAs are oncological diseases. In total, eleven oncological diseases have more than five AAs with NSCLC leading with 21 AAs in total.

**Fig. 6:**
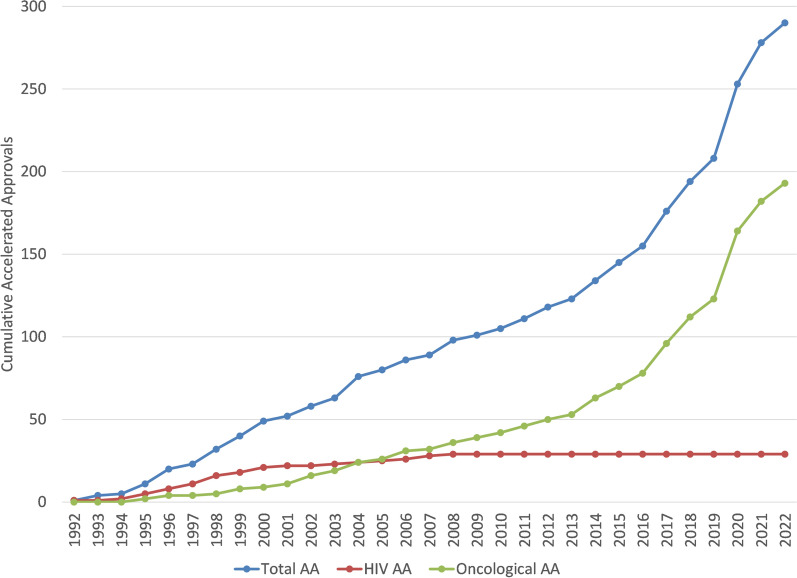
Cumulative AAs by most common AA disease groups

**Fig. 7:**
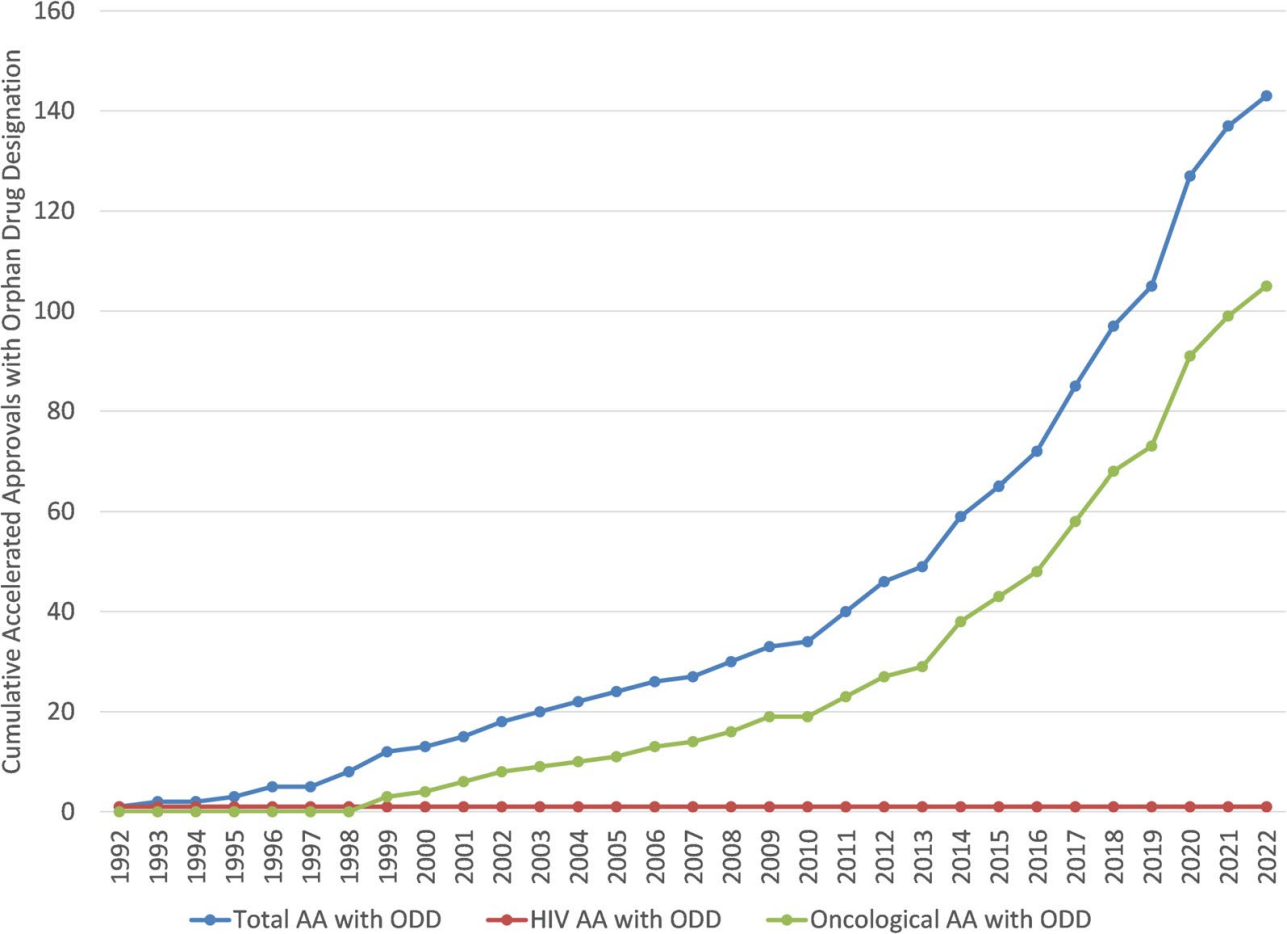
Cumulative AAs with ODD by most common AA disease groups

The increase in AAs targeting oncological diseases has occurred mostly in the recent two decades. The cumulative trajectories of AAs targeting HIV, oncological, and in total across all diseases are depicted in Fig. 6. HIV AAs have plateaued since 2007. Oncological AAs have increased significantly since 2000 and represent the majority of AAs. A similar graphic is displayed in Fig. 7 with AAs that were designated as orphan drugs. This latter figure has a similar pattern as the pattern in Fig. 6 where AAs with ODD are increasing recently and mostly due to drugs targeting oncological diseases.

**Fig. 8:**
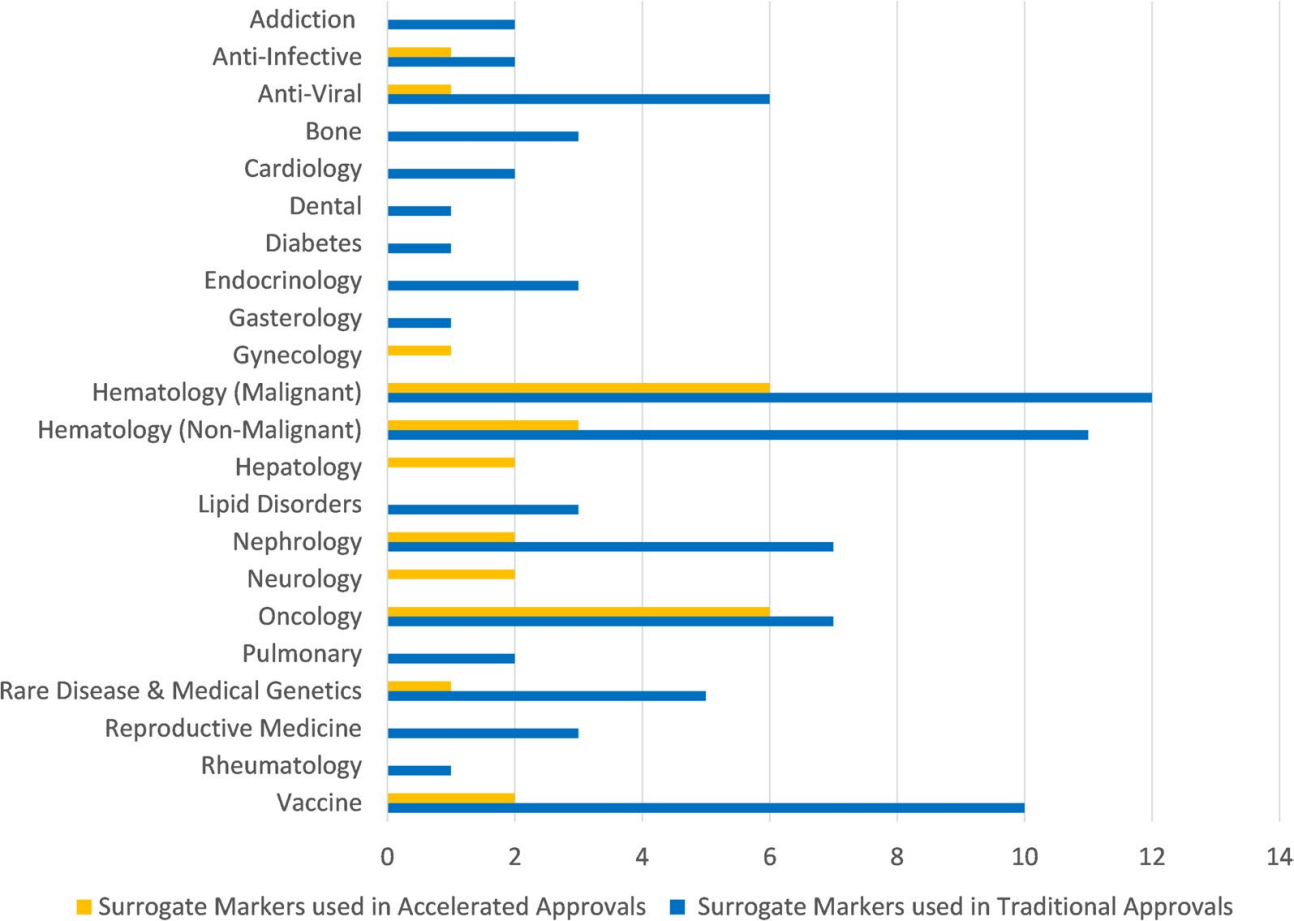
Surrogate markers used in traditional approvals and accelerated approvals by disease area

The count of surrogate markers used in traditional approvals and accelerated approvals are presented in Fig. 8 based on the U.S. FDA’s Adult Surrogate Endpoint Table and assigning a disease to each target patient population. The areas of oncological diseases (oncology and malignant hematology) are the two areas with the greatest number of surrogate markers used in accelerated approvals.

**Fig. 9:**
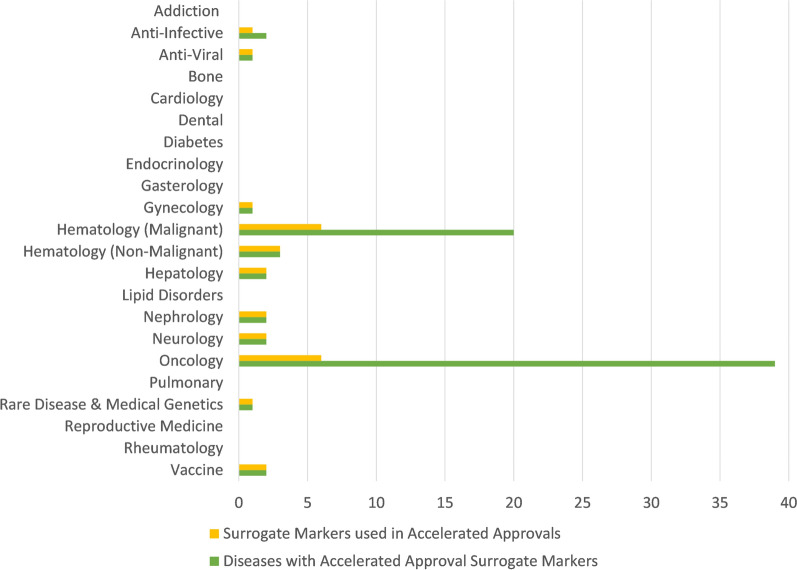
AA Surrogate markers and diseases covered by those markers

The numbers of diseases across which surrogate markers were used in AAs are presented in Fig. 9 alongside the number of surrogate markers used in AAs. The oncological diseases are the only areas that demonstrated significantly more diseases than surrogate markers used in AA, with an average of 5.4 diseases per surrogate marker in the oncological diseases compared to an average of 1.1 diseases per surrogate marker in all other AA. In the non-oncological diseases, the surrogate endpoint of sputum culture conversion to negative was utilized for patients with two lung diseases (pulmonary tuberculosis and Mycobacterium avium complex lung disease). This indicates that disease areas other than oncological diseases have not had significant re-use of surrogate markers in AAs across multiple diseases, suggesting that most non-oncological diseases with therapies utilizing AA have had to establish a novel and/or specific surrogate marker.

One aspect of AAs is the expectation of confirmation of positive benefit-risk profile in subsequent trials or withdrawal of the therapy in cases where negative benefit-risk profile is subsequently observed. Whether positive or negative benefit-risk, the rate of conversion (traditional approval or withdrawn) from AA grouped based on the most common disease groups is tabulated in Table 1 based on the U.S. FDA CDER NDA and Biologics License Applications (BLA) Accelerated Approval Table. The data were assessed as of the end of 2022 by which point 194 AA had been converted (159 to a traditional approval and 35 withdrawn). For example, only AAs that were approved by the end of 2018 were considered for the “Converted within 4 Years?” column since more recent AAs would not yet have four years of data to consider. Conversion times up to five years are considered to be consistent with similar recent analysis and representative of confirmation timelines agreed on by sponsors and the FDA (Shahzad, 2023). In some situations, five years may not be sufficient to fully demonstrate the data required for a traditional approval application. The final column that assesses conversion by the end of 2022 incorporates all AAs approved between 1992 and 2021. For example, all AA for the HIV disease group were approved prior to 2009 and all have been converted by the end of 2022. The “All Others” category (excluding HIV and oncological diseases) constitutes 23% of the accelerated approvals which includes a diverse grouping of conditions and diseases.

**Table 1:** Conversion (Traditional Approval or Withdrawn) Rates of Most Common AA Disease Groups

Disease Group	Number Approved Prior to 2021	Converted within 2 Years of AA?	Converted within 3 Years of AA?	Converted within 4 Years of AA?	Converted within 5 Years of AA?	Converted by End of 2022?
HIV	29	38%	59%	79%	93%	100%
Oncological Diseases	164	15%	32%	48%	61%	66%
All Others	60	7%	9%	23%	31%	64%
Total	253	16%	29%	46%	58%	70%

Similar in logic to Table 1 (where only AA are included that were approved with sufficient elapsed time to be considered for each of the time-specified columns), the rates of conversion of all AAs are tabulated based on decade of AA in Table 2. The entry for the most recent decade for the final column is noted as “N/A” because a high fraction of AA in the most recent decade have not had more than five years elapsed since the AA. The high rates of conversion (98% and 98% for each of the first two rows) for AA prior to 2011 indicate that nearly all of the first 105 AA that were approved have been converted. Given that the recent 2011-2020 trajectories of conversion (within 2 years, withing 3 years, etc.) are consistent with prior time periods, it is plausible that the pattern of high rates of conversion (e.g., 98%) will eventually occur for the recent AA approvals.

**Table 2:** Conversion (Traditional Approval or Withdrawn) Rates of Across All Diseases Based on AA Years

Range of AA Years	Number of AA	Converted within 2 Years of AA?	Converted within 3 Years of AA?	Converted within 4 Years of AA?	Converted within 5 Years of AA?	Converted by End of 2022?
1992-2000	49	13%	27%	45%	59%	98%
2001-2010	56	20%	29%	41%	54%	98%
2011-2020	148	16%	31%	49%	61%	N/A
Total	253	16%	29%	46%	58%	

## Discussion

As presented in Fig. 1, the count of orphan designations is many times larger than the count of orphan designation approvals suggesting that the designation of a therapy for an orphan indication is not a reliable predictor of that therapy being subsequently approved. Further related research could be pursued with regards to the drivers behind the large difference between ODDs and subsequent approvals. The increasing number of orphan designations and approvals in the recent two decades does suggest an investment in the pursuit of orphan-appropriate diseases and/or orphan-appropriate indications (indications falling below the orphan threshold for number of American diagnoses). During that same time period, approved NMEs for indications not approved as orphan designated did not increase, suggesting a preferential increase in investment in pursuit of NMEs for orphan-designated diseases/indications. This data assessment is limited with regards to relevant inferences that can be drawn based on the summary-level assessment conducted without the assessment of individual histories of pursuits of orphan-designated indications.

Of particular note in the data shown in Fig. 2 is the magnitude of the quantity and the recent increase in quantity of orphan drug approvals for oncological diseases (which includes oncological and malignant hematology). In quantity of orphan approvals, the oncological diseases is as large as the next five largest orphan drug disease areas (those displayed in Fig. 2) combined. Almost all of the largest orphan drug disease areas increased significantly in orphan drug approvals in the most recent decade compared to prior decades. Aligned with the general increase in orphan approvals in the most recent decade (shown in Fig. 2), the most recent decade had a higher count of orphan NME approvals than the prior decade as displayed in Fig. 3. These increases suggest significant investment in and availability of novel therapies for patients with orphan-designated diseases and indications. As displayed in Fig. 4, diseases reviewed by the FDA Division of Rare Diseases and Medical Genetics have been the group with the highest fraction of orphan drug NMEs (49%) compared to other disease areas (and particularly higher than the fraction of orphan drug NMEs in oncological diseases). High fractions of NMEs are likely associated with the lack of direct re-use of existing molecules due to a lack of similar diseases/indications. As a group, the non-oncological rare diseases are expected to contain a relatively high fraction of diseases with differentiated etiologies and therefore more likely than average to require NMEs as therapies for orphan-relevant designations (as previously approved therapies/molecules are unlikely to address rare diseases that do not have previously approved drug therapies). In contrast, the oncological disease area (malignant hematology and oncology) has had 32% of orphan drug approvals also as NMEs, suggesting that the balance of 68% are not NMEs. Further related research could be pursued with regards to the extent of novelty of the therapies designated as NMEs.

As can be inferred from the data displayed in Fig. 5 and in Fig. 6, therapies for oncological diseases have become the most frequent applications to successfully use the accelerated approval program with 67% of total accelerated approvals and 73% of the orphan designated accelerated approvals (and in the decade ending at the end of 2022: 84% and 81%, respectively). This is consistent with a recent review of FDA expedited drug development and review programs that showed applications to address oncological diseases represented the majority of recent usage of these programs (Monge, 2022). Once the credibility of surrogate markers is established, then those surrogate markers can sometimes be used for assessing disease progression in similar diseases. This has been highly relevant to oncological diseases where there are often similar etiologies and a surrogate marker can be applicable to many patient populations (e.g. those in various diseases and those segregated based on demographics or clinical markers). This re-use or repeated use of a surrogate marker across different diseases provides both an established surrogate marker and also provides additional evidence regarding the clinical meaningfulness of the surrogate marker which may explain the apparent high rates of willingness to use AA in oncological areas. As shown in the data displayed in Fig. 8 and Fig. 9 oncological diseases are the only areas that has had AA surrogate markers used significantly across different diseases.

Many oncological diseases have significant similarities in etiology which is not often the case in other disease areas. This lack of re-use of similar surrogate markers in most disease areas complicates the practical use of the AA program for non-oncological diseases. Towards reducing this complication, Kakkis et at [7] proposed implementing a Biomarker Qualification Request Process (and an associated scientific framework for qualifying) for new biomarkers and the FDA has recently initiated a Rare Diseases Endpoint Advanced Pilot Program as one of the commitments under the sixth reauthorization of the Prescription Drug User Fee Act (PDUFA VII) [17, 18, 20–23]. The movement towards non-oncological rare disease genetic therapies targeting the underlying cause suggests growing opportunities for use of AA where biomarkers exist on the causal pathway. With appropriate efforts to characterize, these causal biomarkers may provide even greater levels of confidence in using AA for rare genetic diseases [7].

At the disease group level, the data shown in Table 1 suggests that HIV therapies were typically converted (traditional approval or withdrawn) in shorter timeframes than oncological disease therapies and that oncological disease therapies were typically converted in shorter timeframes than all others (diseases that are neither HIV or oncological). Conversion rate of AA is expected to depend on many factors including:

- Speed of progression of clinical outcomes appropriate for assessing benefit-risk profiles
- Ethical and logistical factors relevant to conducting a confirmatory clinical trial
- The size, distribution, and clinical variability of the eligible population
- Changes in treatment options/guidelines following AA approval
- Demonstrated confidence in surrogate marker(s) used
- Clarity of the data resulting from a relevant clinical trial
- Clarity of regulatory communications/expectations.

The FDA Oncology Center of Excellence has described some potential considerations in its management of the on-ramps and off-ramps for the AA program. These potential considerations include planned interim analysis of on-going and/or confirmatory trials to provide information earlier in the AA confirmation process and provide avenues of interaction between sponsors and FDA. [8]. While analysis has shown that starting confirmatory trials prior to approval is associated with a decrease in time to conversion [14],Benjamin, 2023), it should be noted that this and potential enforcement mechanisms should consider the factors in the bulleted list above that influence the time to conversion. An attempt to apply standard requirements across all AAs may be logically inconsistent with the variety of factors observed in practice.

Although the dominant disease group utilizing AA (oncology/HIV/All others) differed significantly over the decades, the summary statistics regarding timeframes for conversion did not. In all three decades, all probabilities of conversion with 2, 3, 4, or 5 years of AA were within 5% absolute of the mean across all decades. Although this assessment is limited by the use of summary statistics (and not histories of development and regulatory review of individual therapies), this temporal consistency at a summary level suggests that the overall patterns to complete traditional approval or withdrawal have continued over the recent decades.

## Conclusions

The history of ODD and AA were quantified and assessed from various perspectives. An increased number of ODDs and orphan NMEs in the most recent decade suggests increased investment in novel treatments targeting diseases infrequently impacting Americans.

The recent ODDs are largely impacted by therapies targeting oncological diseases. Some of these oncological therapies are novel and target relatively narrow patient population while many re-use established surrogate markers for relatively narrow therapeutic indications. In this way, therapies targeting oncological diseases make significant use of both the ODD and/or AA regulatory approaches. The observed increase in ODD and AA after the legislative developments are not necessarily caused by the same drivers for both ODD and AA.

There was only one non-oncological disease area (compared to dozens of oncological diseases) that utilized a surrogate marker in multiple AA for different diseases suggesting one historical difficulty of non-oncology disease areas utilizing the AA regulatory approach (as qualifying a novel surrogate marker for each disease is a complex pursuit).

AA does involve the commitment to perform confirmatory studies that confirm clinical benefit with traditional approval or lead to withdrawal of the product. The fraction of confirmations or withdrawals completed within various timeframes were quantified and compared across disease areas and over time. Therapies for HIV were notably faster to confirm than those for oncology, which, in turn, were notably faster to confirm than other disease areas. Independent of disease area, the timeframes for confirmations were consistent over the recent decades.

## Data Availability

The datasets generated during and/or analysed during the current study are available in the following publicly available repositories: U.S. 17, 18, 20–23. List of Accelerated Approval. Retrieved from https://www.fda.gov/media/88907/download, U.S. 17, 18, 20–23). Orphan Drug Approvals. Retrieved from https://www.accessdata.fda.gov/scripts/opdlisting/oopd/

## Abbreviations

AA: Accelerated Approval
ALL: Acute Lymphoblastic Leukemia
CDER: Center for Drug Evaluation and Research (within FDA)
CML: Chronic Myelogenous Leukemia
CRC: Colorectal Cancer
FDA: United States Food and Drug Administration
HIV: Human Immunodeficiency Virus
MCL: Mantel Cell Lymphona
MM: Multiple Myeloma
MUC: Metastatic Urothelial Carcinoma
NME: New Molecular Entity
NSCLC: Non-Small Cell Lung Cancer
ODA: Orphan Drug Act
ODD: Orphan Drug Designation
RA: Regular Approval
